# Supplementary material for: Heat adaptation of phage T7 under an extended genetic code
Source: Virus Evol. 2021 Dec 1;7(2):veab100. doi: 10.1093/ve/veab100 (PMC8923235; doi:10.1093/ve/veab100)
Supplement: veab100_Supp [file veab100_supp.zip › Sequencing_Analysis.zip!/Sequencing_Analysis/S1/evidence/JC_54.html]

BRESEQ :: Evidence


| Predicted mutation | | | | | | |
| --- | --- | --- | --- | --- | --- | --- |
| evidence | seq id | position | mutation | annotation | gene | description |
| MC JC | NC\_001604.1 | 13,569 | Δ770 bp | intergenic (–/–) | – / – | –/– |

| Missing coverage evidence... | | | | | | | | | | |
| --- | --- | --- | --- | --- | --- | --- | --- | --- | --- | --- |
|  |  |  | seq id | start | end | size | ←reads | reads→ | gene | description |
| \* | \* | ÷ | NC\_001604.1 | 13569 | 14338 | 770 | 4673 [104] | [4] 4561 | *–/–* | –/– |

| New junction evidence | | | | | | | | | | | |
| --- | --- | --- | --- | --- | --- | --- | --- | --- | --- | --- | --- |
|  | | seq id | position | reads (cov) | reads (cov) | score | skew | freq | annotation | gene | product |
| \* | ? | NC\_001604.1 | = 13568 | 104 (0.020) | 4361 (1.060) | 369/586 | 0.2 | 98.8% | intergenic (–/–) | *–/–* | –/– |
|  | ? | NC\_001604.1 | 14339 = | 1 (0.000) |  |  |  |  | intergenic (–/–) | *–/–* | –/– |

Only 100 of 4608 total aligned reads displayed.  

`CTGACAGGATTCTTGATGACTTTCCAGACGACTACGAGAAGTTTCGCTGGAGAGTCCCATTCTAATACGACTCACTAAAGGAGACACACCATGTTCAAACTGATTAAGAAGTTAGGCCAACTGCTGGTTCGTATGTACAACGTGGAAGCCAAGCGACTGAACGATGAGGCTCGTAAAGAGGCCACACAGTCACGCGCTCTGGCGATTCGCTCCAACGAACTGGCTGACAGTGCATCCACTAAAGTTACCGAGGCTGCCCGTGTGGCAAACCAAGCTCAACAGCTTTCCAAATTCTTTGAGTAATC‑A‑A‑‑‑‑‑‑‑‑‑‑‑‑‑‑‑‑‑‑‑‑‑‑‑‑‑‑‑‑‑‑‑‑‑‑‑‑‑‑‑‑‑‑‑‑‑‑‑‑‑‑‑‑‑‑‑‑‑‑‑‑‑‑‑‑‑‑‑‑‑‑‑‑‑‑‑‑‑‑‑‑‑‑‑‑‑‑‑‑‑‑‑‑‑‑‑‑‑‑‑‑‑‑‑‑‑‑‑‑‑‑‑‑‑‑‑‑‑‑‑‑‑‑‑‑‑‑‑‑‑‑‑‑‑‑‑‑‑‑‑‑‑‑‑‑‑‑‑‑‑‑‑‑‑‑‑‑‑‑‑‑‑‑‑‑‑‑‑‑‑‑‑‑‑‑‑‑‑‑‑‑‑‑‑‑‑‑‑‑‑‑‑‑‑‑‑‑‑‑‑‑‑‑‑‑‑‑‑‑‑‑‑‑‑‑‑‑‑‑‑‑‑‑‑‑‑‑‑‑‑‑‑‑‑‑‑‑‑‑‑‑‑‑‑‑‑‑‑‑‑‑‑‑‑‑‑‑‑‑‑‑‑‑‑‑‑‑‑‑‑‑‑‑‑‑‑‑‑‑‑‑‑‑‑‑‑‑‑‑‑‑‑‑‑‑‑‑‑‑‑‑‑  >  NC_001604.1/13262‑13568`  
`‑‑‑‑‑‑‑‑‑‑‑‑‑‑‑‑‑‑‑‑‑‑‑‑‑‑‑‑‑‑‑‑‑‑‑‑‑‑‑‑‑‑‑‑‑‑‑‑‑‑‑‑‑‑‑‑‑‑‑‑‑‑‑‑‑‑‑‑‑‑‑‑‑‑‑‑‑‑‑‑‑‑‑‑‑‑‑‑‑‑‑‑‑‑‑‑‑‑‑‑‑‑‑‑‑‑‑‑‑‑‑‑‑‑‑‑‑‑‑‑‑‑‑‑‑‑‑‑‑‑‑‑‑‑‑‑‑‑‑‑‑‑‑‑‑‑‑‑‑‑‑‑‑‑‑‑‑‑‑‑‑‑‑‑‑‑‑‑‑‑‑‑‑‑‑‑‑‑‑‑‑‑‑‑‑‑‑‑‑‑‑‑‑‑‑‑‑‑‑‑‑‑‑‑‑‑‑‑‑‑‑‑‑‑‑‑‑‑‑‑‑‑‑‑‑‑‑‑‑‑‑‑‑‑‑‑‑‑‑‑‑‑‑‑‑‑‑‑‑‑‑‑‑‑‑‑‑‑‑‑‑‑‑‑‑‑‑‑‑‑‑‑‑‑‑‑‑‑‑‑‑‑‑‑‑‑‑‑‑‑‑‑‑‑‑‑‑‑‑‑taatc‑a‑aT‑AGGAGAAATCAATATGATCGTTTCTGACATCGAAGCTAACGCCCTCTTAGAGAGCGTCACTAAGTTCCACTGCGGGGTTATCTACGACTACTCCACCGCTGAGTACGTAAGCTACCGTCCGAGTGACTTCGGTGCGTATCTGGATGCGCTGGAAGCCGAGGTTGCACGAGGCGGTCTTATTGTGTTCCACAACGGTCACAAGTATGACGTTCCTGCATTGACCAAACTGGCAAAGTTGCAATTGAACCGAGAGTTCCACCTTCCTCGTGAGAACTGTATTGACACCCTTGTGTTGTCAC  >  NC_001604.1/14339‑14638`  

                                                                                                                                                                                                                                                                                                                                                                                                                                                                                                                                                                                                                                    
 `TGACAGGATTCTTGATGACTTTCCAGACGACTACGAGAAGTTTCGCTGGAGAGTCCCATTCTAATACGACTCACTAAAGGAGACACACCATGTTCAAACTGATTAAGAAGTTAGGCCAACTGCTGGTTCGTATGTACAACGTGGAAGCCAAGCGACTGAACGATGAGGCTCGTAAAGAGGCCACACAGTCACGCGCTCTGGCGATTCGCTCCAACGAACTGGCTGACAGTGCATCCGCTAAAGTTACCGAGGCTGCCCGTGTGGCAAACCAAGCTCAACAGCTTTCCAAATTCTTTGAGTA                                                                                                                                                                                                                                                                                                                      >  1:267622/1‑301`  
       `acagCTTGATGACTGTCCAGACGACTACGAGAAGTTTCGCTGGAGAGTCCCATTCTAATACGACTCACTAAAGGAGACACACCATGTTCAAACTGATTAAGAAGTTAGGCCAACTGCTGGTTCGTATGTACAACGTGGAAGCCAAGCGACTGAACGATGAGGCTCGTAAAGAGGCCACACAGTCACGCGCTCTGGCGATTCGCTCCAACGAACTGGCTGACAGTGCATCCACTAAAGTTACCTAGGCTGCCCGTGTGGCAAACCAAGCTCAACAGCTTTCCAAATTCTTTGAGTAATC‑A‑AT                                                                                                                                                                                                                                                                                                              <  1:164997/297‑1`  
          `TCTTGATTACTTTCCAGACGACTACGAGAAGTTTCGCTGGAGAGTCCCATTCTAATACGACTCACTAAAGGAGACACACCATGTTCAAACTGATTAAGAAGTTAGGCCAACTGCTGGTTCGTATGTACAACGTGGAAGCCAAGCGACTGAACGATGAGGCTCGTAAAGAGGCCACACAGTCACGCACTCTGGCGATTCGCTCCAACGAACTGGCTGACAGTGCATCCACTAAAGTTACCGAGGCTGCCCGTGTGGCAAACCAAGCTCAACAGCTTTCCAAATTCTTTGAGTAATC‑A‑AT‑AGG                                                                                                                                                                                                                                                                                                          <  1:110685/301‑1`  
          `TCTTGATGACTTTCCAGACGACTACGAGAAGGGTCGCTGGAGAGTCCGATTCTAATACGACTCACTAAAGGAGACACACCATGTTCAAACTGATTAAGAAGTTAGGCCAACTGCTGTTTCGTATGTACAACGTGGAAGCCAAGCGACTGAACGATGAGGCTCGTAAAGAGGCCACACAGTCACGCGCTCTGGCGATTCGCTCCAACGAACTGGCTGACAGTGCATCCACTAAAGTTACCGAGGCTGCCCGTGTGGCAAACCAAGCTCAACAGCTTTCCAAATTCTTTGAGTAATC‑A‑AT‑AGG                                                                                                                                                                                                                                                                                                          <  1:315264/301‑1`  
                            `CGACTACGAGAAGTTTCGCTGGAGAGTCCCATTCTAATACGACTCACTAAAGGAGACACACCATGTTCAAACTGATTAAGAAGTTAGGCCAACTGCTGGTTCGTATGTACAACGTGGAAGCCAAGCGACTGAACGATGAGGCTCGTAAAGAGGCCACACAGTCACGCGCTCTGGCGATTCGCTCCAACGAACTGGCTGACAGTTCATCCACTAAAGTTACCGAGGCTGCCCGTGTGGCAAACCAAGCTCAACAGCTTTCCAAATTCTTTGCGTAATC‑A‑AT‑AGGctgtctcttatacacatc                                                                                                                                                                                                                                                                                        >  1:333728/1‑283`  
                               `CTACGAGAAGTTTCGCTGGAGAGTCCCATTCTAATACGACTCACTAAAGGAGACACACCATGTTCAAACTGATTAAGAAGTTAGGCCAACTGCTGGTTCGTATGTACAACGTGGAAGCCAAGCGACTGAACGATGAGGCTCGTAAAGAGGCCACACAGTCACGCGCTCTGGCGATTCGCTCCAACGAACTGGCTGACAGTGCATCCACTAAAGTTACCGAGGCTGCCCGTGTGGCAAACCAAGCTCAACAGCTTTCCAAATTCTTTGAGTAATCAA‑AC‑AGGAGAAATCAATATGATCGTTT                                                                                                                                                                                                                                                                                      >  1:100160/1‑301`  
                                   `GAGAAGTTTCGCTGGAGAGTCCCATTCTAATACGACTCACTAAAGGAGACACACCATGTTCAAACTGATTAAGAAGTTAGGCCAACTGCTGGTTCGTATGTACAACGTGGAAGCCAAGCGACTGAACGATGAGGCTCGTAAAGAGGCCACACAGTCACGCGCTCTGGCGATTCGCTCCAACGAACTGGCTGACAGTGCATCCACTAAAGTTACCGAGGCTGCCCGTGTGGCAAACCAAGCTCAACAGCTTTCCAAATTCTTTGAGTAATC‑A‑AT‑AGGAGAAATCAATATGATCGTTTCTGAC                                                                                                                                                                                                                                                                                 <  1:536260/301‑1`  
                                            `CGCTGGAGAGTCCCATTCTAATACGACTCACTAAAGGAGACACACCATGTTCAAACTGATTAAGAAGTTAGGCCAACTGCTGGTTCGTATGTACAACGTGGAAGCCAAGCGACTGAACGATGAGGCTCGTAAAGAGGCCACACAGTCACGCGCTCTGGCGATTCGCTCCAACGAACTGGCTGACAGTGCATCCACTAAAGTTACCGAGGCTGCCCGTGTGGCAAACCAAGCTCAACAGCTTTCCAAATTCTTTGAGTAATCAA‑AC‑AGGAGAAATCAATATGATCGTTTCTGACATCGAAGC                                                                                                                                                                                                                                                                         >  1:65375/1‑301`  
                                                         `ggagatgtgtataagagacagAGGAGACACACCATGTTCAAACTGATTAAGAAGTTAGGCCAACTTCTGGTTCGTATGTACAACGTGGAAGCCAATCGACTGAACGATGAGGCTCGTAAAGAGGCCACACAGTCACGCGCTCTGGCGATTCGCTCCAACGAACTGGCTGACAGTGCATCCACTAAAGTTACCGAGGCTGCCCGTGTGGCAAACCAAGCTCAACATCTTTCCAAATTCTTTGAGTAATT‑A‑AT‑AGGAGAAATCAATATGATCGTTTCTGACATCGAAGCTAACGCCCTCTTAG                                                                                                                                                                                                                                                           <  1:375846/280‑1`  
                                                           `tttcAATACGACTCACTAAAGGAGACACGCCATGTTCAAACTGATTAAGAAGTTAGGCCAACTGCTAGTTCGTATGTACAACGTGGAAGCCAAGCGACTGAACGATGAGGCTCGTAAAGAGGCCACACAGTCACGCGCTCTGGCGATTCGCTCCAACGAACTGGCTGACAGTGCATCCACTAAAGTTACCGAGGCTGCCCGTGTGGCAAACCAAGCTCAACAGCTTTCCAAATTCTTTGAGTAATC‑A‑AT‑AGGAGAAATCAATATGATCGTTTCTGACATCGAAGCTAACGCCCTCTTAGAG                                                                                                                                                                                                                                                         <  1:585145/297‑1`  
                                                           `ttaTAATACGACTCACTAAAGGAGACACACCATGTTCAAACTGATTAAGAAGTTAGGCCAACTGCTTGTTCGTATGTACAACGTGGAAGCCAAGCGACTGAACGATGAGGCTCGTAAAGAGGCCACACAGTCACGCGCTCTGGCGATTCGCTCCAACGAACTGGCTGACAGTGCATCCACTAAAGTTACCGAGGCTGCCCGTGTGGCAAACCAAGCTCAACAGCTTTCCAAATTCTTTGAGTAATC‑A‑AT‑AGGAGAAATCAATATGATCGTTTCTGACATCGAAGCTAACGCCCTCTTAGAG                                                                                                                                                                                                                                                         <  1:577546/298‑1`  
                                                           `taCTAAGACGACCAACTAAAGGAGACACACCATGTTAAAACTGATTAAGAAGTGAGGCCAACTGCTGGTGCGTATGTACAACGTGGAAGCCAAGCGACTGAACGATGAGGCTCGTAAAGAGGCCACACAGTCACGCGCTCTGGCGATTCGCTCCAACGAACTGGCTGACAGTGCATCCACTAAAGTTACCGAGGCTGCCCGTGTGGCAAACCAAGCTCAACAGCTTTCCAAATTCTTTGAGTAATC‑A‑AT‑AGGAGAAATCAATATGATCGTTTCTGACATCGAAGCTAACGCCCTCTTAGAG                                                                                                                                                                                                                                                         <  1:231728/299‑1`  
                                                           `aaggAATACTACTCACAAAAGGAGACAAACCAAGTTCAAACTGAGTAAGAAGGGAGGCCAAATGCGGGTTCGTATGTACAACCGGGAAGCCAAGCGACTGAACGATGAGGCGCGGAAAGAGGCCACACAGTCACGCGCTCTGGCGATTCGGGCCAACGAACTGGCTGACAGGGCATCCACTAAAGTGACCGAGGCGGCCCGTGTGGCAAAACAAGCTCAACAGCTTTCCAAATTCTGTGAGTAAGC‑A‑AT‑AGGAGAAATCAATATGATCGTTTATGACATCGAAGCTAACGCCCTCTTAGAG                                                                                                                                                                                                                                                         <  1:395347/297‑1`  
                                                           `TTCTAATATGACTCACTAAAGGAGACACACCATGTTCAAACTGATTAAGAAGTTAGGCCAACTGCTGGTTCGTATGTACAACGTGGAAGCCAAGCGACTGAACGATGAGGCTCGTAAAGAGGCCACACAGTCACGCGCTCTGGCGATTCGCTCCAACGAACTGGCTGACAGTGCATCCACTAAAGTTACCGAGGCTGCCCGTGTGGCAAACCAAGCTCAACAGCTTTCCAAATTCTTTGAGTAATCAA‑AC‑AGGAGAAATCAATATGATCGTTTCTGACATCGAAGCTAACGCCCTCTTAGA                                                                                                                                                                                                                                                          >  1:297901/1‑301`  
                                                           `TTCTAATACGACTCACTAAAGGCGACACACCATGTTCAAACTGATTAAGAAGTTAGGCCAACTGCTGGTTCGTATGTACAACGTGGAAGCCAAGCGACTGAACGATGAGGCTCGTAAAGAGGCCACACAGTCACGCGCTCTGGCGATTCGCTCCAACGAACTGGCTGACAGTGCATCCACTAAAGTTACCGAGGCTGCCCGTGTGGCAAACCAAGCTCAACAGCTTTCCAAATTCTTTGAGTAAAC‑A‑AT‑AGGAGAAATCAATATGATCGTTTCTGACATCGAAGCTAACGCCCTCTTAGAG                                                                                                                                                                                                                                                         <  1:380794/301‑1`  
                                                           `TTCTAATACGACTCACTAAAGGAGACACACCATGTTCAAACTGATTAAGAAGTTAGGCCAACTGCTGGTTCGTATGTACAACGTGGAAGCCAAGCGACTGAACGATGAGGCTCGTAAAGAGGCCACACAGTCACGCGCTCTGGCGATTCGCTCCAACGAACTGGCTGACAGTGCATCCACTAAAGTTACCGAGGCTGCCCGTGTGGCAAACCAAGCTCAACAGCTTTCCAACTTCTTTGAGTAATC‑A‑AT‑AGGAGAAATCAATATGATCGTTTCTGACATCGAAGCTAACGCCCCCTTAGAG                                                                                                                                                                                                                                                         >  1:208830/1‑301`  
                                                           `TTCTAATACGACTCACTAAAGGAGACACACCATGTTCAAACTGATTAAGAAGTTAGGCCAACTGCTGGTTCGTATGTACAACGTGGAAGCCAAGCGACTGAACGATGAGGCTCGTAAAGAGGCCACACAGTCACGCGCTCTGGCGATTCGCTCCAACGAACTGGCTGACAGTGCATCCACTAAAGTTACCGAGGCTGCCCGTGTGGCAAACCAAGCTCAACAGCTTTCCAAATTCTTTGAGTAATCAA‑AC‑AGGAGAAATCAATATGATCGTTTCTGACATCGAAGCTAACGCCCTCTTAGA                                                                                                                                                                                                                                                          >  1:406613/1‑301`  
                                                           `TTCTAATACGACTCACTAAAGGAGACACACCATGTTCAAACTGATTAAGAAGTTAGGCCAACTGCTGGTTCGTATGTACAACGTGGAAGCCAAGCGACTGAACGATGAGGCTCGTAAAGAGGCCACACAGTCACGCGCTCTGGCGATTCGCTCCAACGAACTGGCTGACAGTGCATCCACTAAAGTTACCGAGGCTGCCCGTGTGGCAAACCAAGCTCAACAGCTTTCCAAATTCTTTGAGTAATCAA‑AC‑AAGAGAAATCAATATGATCGTTTCTGACATCGAAGCTAACGCCCTCTTAGA                                                                                                                                                                                                                                                          >  1:196893/1‑301`  
                                                           `TTCTAATACGACTCACTAAAGGAGACACACCATGTTCAAACTGATTAAGAAGTTAGGCCAACTGCTGGTTCGTATGTACAACGTGGAAGCCAAGCGACTGAACGATGAGGCTCGTAAAGAGGCCACACAGTCACGCGCTCTGGCGATTCGCTCCAACGAACTGGCTGACAGTGCATCCACTAAAGTTACCGAGGCTGCCCGTGTGGCAAACCAAGCTCAACAGCTTTCCAAATTCTTTGAGTAATC‑A‑AT‑AGGAGAAATCAATATGATCGTTTCTGACATCGAAGCTAACGCCCTCTTAGAG                                                                                                                                                                                                                                                         <  1:45333/301‑1`  
                                                           `TTCTAATACGACTCACTAAAGGAGACACACCATGTTCAAACTGATTAAGAAGTTAGGCCAACTGCTGGTTCGTATGTACAACGTGGAAGCCAAGCGACTGAACGATGAGGCTCGTAAAGAGGCCACACAGTCACGCGCTCTGGCGATTCGCTCAAACGAACTGGCTGACAGTGCATCCACTAAAGTTACCGAGGCTGCCCGTGTGGCAAACCAAGCTCAACAGCTTTCCAAATTCTTTGAGTAATC‑A‑AT‑AGGAGAAATCAATATGATCGTTTCTGACATCGAAGCTAACGCCCTCTTAGAt                                                                                                                                                                                                                                                         <  1:93/301‑2`  
                                                           `TTCTAATACGACTCACTAAAGGAGACACACCATGTTCAAACTGATTAAGAAGTTAGGCCAACTGCTGGTTCGTATGTACAACGTGGAAGCCAAGCGACTGAACGATGAGGCTCGTAAAGAGGCCACACAATCACGCGCTCTGGCGATTCGCTCCAACGAACTGGCTGACAGTGCATCCACTAAAGTTACCGAGGCTGCCCGTGTGGCAAACCAAGCTCAACAGCTTTCCAAATTCTTTGAGTAATC‑A‑AT‑AGGAGAAATCAATATGATCGTTTCTGACATCGAAGCTAACGCCCTCTTAGAG                                                                                                                                                                                                                                                         <  1:569359/301‑1`  
                                                           `TTCTAATACGACTCACTAAAGGAGACACACCATGTTCAAACTGATTAAAAAGTTAGGCCAACTGCTGGTTCGTATGTACAACGTGGAAGCCAAGCGACTGAACGATGAGGCTCGTAAAGAGGCCACACAGTCACGCGCTCTGGCGATTCGCTCCAACGAACTGGCTGACAGTGCATCCACTAAAGTTACCGAGGCTGCCCGTGTGGCAAACCAAGCTCAACAGCTTTCCAAATTCTTTGAGTAATCAA‑AC‑AGGAGAAATCAATATGATCGTTTCTGACATCGAAGCTAACGCCCTCTTAGA                                                                                                                                                                                                                                                          >  1:321703/1‑301`  
                                                            `aagaagagacagcaaTAAAGGAGACACACCATGTTCAACCTGATTAAGAAGTTAGGCCAACTGCTGGTTCGTATTTACAACGTGGAAGCCAAGCGACTGAACGATGAGGCTCGTAAAGATGCCACACAGTAACGCGCTCTGGCGATTCGCTCCAACGAACTGGCTGACAGTGCATCCACTAAAGTTACCGAGGCTGCCCGTGTGGCAAACCAAGCGCAACAGCTTTCCAAATTCTTTGAGTAATCAA‑AC‑AGGAGAAATCAATATGATCGTTTCTGACATCGAAGCTAACGCCCTCTTAGAG                                                                                                                                                                                                                                                         <  1:203604/286‑1`  
                                                            `TCTAATACGACTCACTAAAGGAGACACACCATGTTCAAACTGATTAAGAAGTTAGGCCAACTGCTGGTTCGTATGTACAACGTGGAAGCCAAGCGACTGAACGATGAGGCTCGTAAAGAGGCCACACAGTCACGCGCTCTGGCGATTCGCTCCAACGAACTGGCTGACAGTGCATCCACTAAAGTTACCGAGGCTGCCCGTGTGGCAAACCAAGCTCAACAGCTTTCCAAATTCTTTGAGTAATCAA‑AC‑AGGAGAAATCAATATGATCGTTTCTGACATCGAAGCTAACGCCCTCTTAGAG                                                                                                                                                                                                                                                         <  1:122574/301‑1`  
                                                            `TCTAAGACGACTCACTAAAGGAGACACACCATGTTCAATCTGATTAAGAAGTTAGGCCAACTGCTGGTTCGTATGTACAACGTGGAAGCCAAGCGGCTGAACGATGAGGCTCGTAAAGAGGCAACACAGTCACGCGCTCTGGCGATTCGCTCCAACGAACTGGCTGACAGTGCATCCACTAAAGTTACCGAGGCTGCCCGTGTGGCAAACCAAGCTCAACAGCTTTCCAAATTCTTTGAGTAATCAA‑AC‑AGGAGAAATCAATATGATCGTTTCTGACATCGAAGCTAACGCCCTCTTAGAG                                                                                                                                                                                                                                                         <  1:56253/301‑1`  
                                                             `tTAATACGACTCACTAAAGGAGAAACACCAAGTTCAAACTGATTAAGAAGTTAGGCCAACTGCTGGGTCGTATGTACAACGTGGAAGCCAAGCGACTGAACGATGAGGCTCGTAAAGAGGCCAAACAGTCACGCGCTCTGGCGATTCGCTCCAACGAACTGGCTGACAGTGCATCCACTAAAGTTACCGAGGCTGCCCGTGTGGCAAACCAAGCTCAACAGCTTTCCAAATTCTTTGAGTAATC‑A‑AT‑AGGAGAAATCAATATGATCGTTTCTGACATCGAAGCTAACGCCCTCTTAGAGAG                                                                                                                                                                                                                                                       <  1:49487/300‑1`  
                                                                   `CGACTCACTAAAGGAGACACACCATGTTCAAACTGATTAAGAAGTTAGGCCAACTGCTGGTTCGTATGTACAACGTGGAAGCCAAGCGACTGAACGATGAGGCTCGTAAAGAGGCCACACAGTCACGCGCTCTGGCGATTCGCTCCAACGAACTGGCTGACAGTGCATCCACTAAAGTTACCGAGGCTGCCCGTGTGGCAAACCAAGCTCAACAGCTTTCCAAATTCTTTGAGTAATCAA‑AC‑AGGAGAAATCAATATGATCGATTCTGACATCGAAGCTAACTCCATCTTCGAGAGCGgcc                                                                                                                                                                                                                                                  >  1:129671/1‑298`  
                                                                     `ACTCACTAAAGGAGACAAACCAGGTTCAAACTGATTAAGAAGTTAGGCCAACTGCTGGTTCGTATGTACAACGTGGAAGCCAAGCGACTGAACGATGAGGCTCGTAAAGAGGCCACACAGTCACGCGCTCTGGCGATTCGCTCCAACGAACTGGCTGACAGTGCATCCACTAAAGTTACCGAGGCTGCCCGTGTGGCAAACCAAGCTCAACAGCTTTCCAAATTCTTTGAGTAATC‑A‑AT‑AGGAGAAATCAATATGATCGTTTCTGACATCGAAGCTAACGCCCTCTTAGAGAGCGTCACTA                                                                                                                                                                                                                                               <  1:86112/301‑1`  
                                                                        `CACTAAAGGAGACACACCATGTTCAAACTGATTAAGAAGTTAGGCCAACTGCTGGTTCGTATGTACAACGTGGAAGCCAAGCGACTGAACGATGAGGCTCGTAAAGAGGCCACACAGTCACGCGCTCTGGCGATTCGCTCCCACGAACTGGCTGACAGTGCATACACTAAAGTTACCGAGGCTGCCCGTGTGGCAAACCAAGCTCAACAGCTTTACAAATTCTTTGAGTAATC‑A‑AT‑AGGAGAAATCAATATGATCGTTTCTGACATCGAAGCTAACGCCCTCTTCGAGAGCGCCACTAAGT                                                                                                                                                                                                                                            >  1:115880/1‑301`  
                                                                        `CACTAAAGGAGAAACACCAAGTTCAAACTGATTAAGAAGTTAGGCCAACTGCTGGTTCGTATGTACAACGTGGAAGCCAAGCGACTGAACGATGAGGCTCGTAAAGAGGCCACACAGTCACGCGCTCTGGCGATTCGCTCCAACGAACTGGCTGACAGTGCATCCACTAAAGTTACCGAGGCTGCCCGTGTGGCAAACCAAGCTCAACAGCTTTCCAAATTCTTTGAGTAATC‑A‑AT‑AGGAGAAATCAATATGATCGTTTCTGACATCGAAGCTAACGCCCTCTTAGAGAGCGTCACTAAGT                                                                                                                                                                                                                                            <  1:304785/301‑1`  
                                                                          `CTAAAGGAGACACACCATGTTCAAACTGATTAAGAAGTTAGGCCAACTGCTGGTTCGTATGTACAACGTGGAAGCCAAGCGACTGAACGATGAGGCTCGTAAAGAGGCCACACAGTCACGCGCTCTAGCGATTCGCTCCAACGAACTGGCTGACAGTGCATCCACTAAAGTTACCGAGGCTGCCCGTGTGGCAACCCAAGCTCAACAGCTTTCCAAATTCTTTGAGTAATC‑A‑AT‑AGGAGAAATCAATATGATCGTATCTGACATCGAAGCTAACGCCCTCTTAGATAGCGTCACTActgtc                                                                                                                                                                                                                                          >  1:601859/1‑296`  
                                                                            `AAAGGAGACACACCATGTTCAAACTGATTAAGAAGTTAGGCCAACTGCTGGTTCGTATGTACAACGTGGAAGCCAAGCGACTGAACGATGAGGCTCGTAAAGAGGCCACACAGTCACGCGCTCTGGCGATTCGCTCCAACGAACTGGCTGACAGTGCATCCACTAAAGTTACCGAGGCTGCCCGTGTGGCAAACCAAGCTCAACAGCTTTCCAAATTCTTTGAGTAATC‑A‑AT‑AGGAGAAATCAATATGATCGTTTCTGACCTCGAAGCTAACGCCCTCTTAGAGAGCGTCACTAAGTTCtc                                                                                                                                                                                                                                        >  1:659869/1‑299`  
                                                                               `GGAGACACACCATGTTCAAACTGATTAAGAAGTTAGGCCAACTGCTGGTTCGTATGTACAACGTGGAAGCCAAGCGACTGAACGATGAGGCTCGTAAAGAGGCCACACAGTCACGCGCTCTGGCGATTCGCTCCAACGAACTGGCTGACAGTGCATCCACTAAAGTTACCGAGGCTGCCCGTGTGGCAAACCAAGCCCAACAGCTTTCCAAATTCTTTGAGTAATCAA‑AC‑AAGAGAAATCAATATGATCGTTTATGACATCGAAGCTAAAGCACTCTTAGAGctgtctcgtatacacatct                                                                                                                                                                                                                                      >  1:630974/1‑282`  
                                                                                    `aACACCATGTTCAAACTGATTAAGAAGTTAGGCCAACTGCTGGTTCGTATGTACAACGTGGAAGCCAAGCGACTGAACGATGAGGCTCGTAAAGAGGCCACACAGTCATGCGCTCTGGCGATTCGCTCCAACGAACTGGCTGACAGTGCATCCACTAAAGTTACCGAGGCTGCCCGTGTGGCAAACCAAGCTCAACAGCTTTCCAAATTCTTTGAGTAATC‑A‑AT‑AGGAGAAATCAATATGATCGTTTCTGACATCGAAGCTAACGCCCTCTTAGAGAGCGTCACTAAGTTCCACTGCGGGG                                                                                                                                                                                                                                <  1:273091/300‑1`  
                                                                                         `CATGTTCAAACTGATTAAGAACTTAGGCCAACTGCTGGTTCGTATGTACAACGTGGAAGCCAAGCGACTGAACGATGAGGCTCGTAAAGAGGCCACACAGTCACGCGCTCTGGCGATTCGCTCCAACGAACTGGCTGACAGTGCATCCACTAAAGTTACCGAGGCTGCCCGTGTGGCAAACCAAGCTAAACAGCTTTCCAAATTCTTTGAGTAATC‑A‑AT‑AGGAGAAATCAATATGATCGTTTCTGACATCGAAGCTAACGCCCTCTTAGAGAGCGTCACTAAGTTCCACTGCGGGGTTATC                                                                                                                                                                                                                           <  1:440577/301‑1`  
                                                                                          `ATGTTCAAACTGATTAAGAAGTTAGGCCAACTGCTGGTTCGTATGTACAACGTGGAAGCCAAGCGACTGAACGATGAGGCTCGTAAAGAGGCCACACAGTCACGCGCTCTGGCGATTCGCTCCAACGAACTGGCTGACAGTGCATCCACTAACGTTACCGCGGCTGCCCGTGTGGCAAACCCAGCTCCACAGCTTTCCAACTTCTTTGAGTAATC‑A‑ATCAGGACAAACCATTATGATCGTTTCTGACATCGAAGCTAACGCCCTCTTCGAGAGCGTCACTAAGTTCCACTGCGGGTCTATC                                                                                                                                                                                                                           >  1:243871/1‑301`  
                                                                                           `TGTTCAAACTGATTAAGAAGTTAGGCCAACTGCTGGTTCGTATGTACAACGTTGAAGCCAAGCGACTGAACGATGAGGCTCGTAAAGAGGCCACACAGTCACGCGCTCTGGCGATTCGCTCCAACGAACTGGCTGACAGTGCATCCACTAAAGTTACCGAGGCTGCCCGTGTGGCAAACCAAGCTCAACAGCTTTCCAAATTCTTTGAGTAATC‑A‑AT‑AGGAGAAATCAATATGATCGTTTCTGACATCGAAGCTAACGCCCTCTTAGAGAGCGTCACTAAGTTCCACTGCGGGGTTATCTA                                                                                                                                                                                                                         <  1:353158/301‑1`  
                                                                                            `GTTCAAACTGATTAAGAAGTTAGGCCAACTGCTGGTTCGTATGTACAACGTGGAAGCCAAGCGACTGAACGATGAGGCTCGTAAAGAGGCCACACAGTCACGCGCTCTGGCGATTCGCTCCAACGAACTGGCTGACAGTGCATCCACTAAAGTTACCGAGGCTGCCCGTGTGGCAAACCAAGCTCAACAGCTTTCCAAATTCTTTGAGTAATC‑A‑AT‑AGGAGAAATCAATATGATCGTTTCTGACATCGAAGCTAACGCCCTCTTAGAGAGCGTCACTAAGTTCCACTGCGGGGTTATCTAC                                                                                                                                                                                                                        >  1:310466/1‑301`  
                                                                                               `caatgagtTTAGGAAGTTAGGCCAACTGCTGTCACATATGAACAACGTGGAAGCCAACGCACTGTTCGATGCGGTTAGTAAAGATGCAACACAGTCACGCGATTTGGCGATTCGCTGCAACGAACTGGCTGACAGTGCCTCCTCTAAAGTTACCGGGGCTGCCCGCGTGGCAAACCATGCTCAAAAGCTTTCCAAATTCTTTGAGTAATC‑A‑AA‑AGGAGAAATCAATATGATCGTTTCTGACATCGAAGCTAACGCCCTCTTAGAGAGCGTCACTAAGTTCCACTGCGGGGTTATCTACGAC                                                                                                                                                                                                                     <  1:465232/293‑1`  
                                                                                                   `CTGATTAAGAAGTTAGGCCAACTGCTGGTTCGTATGTACAACGTGGAAGCCAAGCGACTGAACGATGAGGCTCGTAAAGAGGCCACACAGTCACGCGCTCTGGCGATTCGCTCCAACGAACTGGCTGACAGTGCATCCACTAAAGTTACCGAGGCTGCCCGTGTGGCAAACCAAGCTCAACAGCTTTCCAAATTCTTTGAGTAATC‑A‑AT‑AGGAGAAATCAATATGATCGTTTCTGACATCGAAGCTAACGCCCTCTTAGAGAGCGTCACTAAGTTCCACTGCGGGGTTATCTACGACTACT                                                                                                                                                                                                                 >  1:340235/1‑301`  
                                                                                                             `AGTTAGGCCAACTGCTGGTTCGTATGTACAACGTGGAAGCCAAGCGACTGAACGATGAGGCTCGTAAAGAGGCCACACAGTCACGCGCTCTGGCGATTCGCTCCAACGAACTGGCTGACAGTGCATCCACTAAAGTTACCGAGGCTGCCCGTGTGGCAAACCAAGCTCAACAGCTTTCCAAATTCTTTGAGTAATC‑A‑AT‑AGGAGAAATCAATATGATCGTTTCTGACATCGAAGCTAACGCCCTCTTAGAGAGCGTCACTAAGTTCCACTGCGGGGTTATCTACGACTACTCCACCtgtct                                                                                                                                                                                                       >  1:471938/1‑296`  
                                                                                                             `AGTTAGGCCAACTGCTGGTTCGTATGTACAACGTGGAAGCCAAGCGACTGAACGATGAGGCTCGTAAAGAGGCCACACAGTCACGCGCTCTGGCGATTCGCTCCAACGAACTGGCTGACAGTGCATCCACTAAAGTTACCGAGGCTGCCCGTGTGGCAAACCAAGCTCAACAGCTTTCCAAATTCTTTGAGTAATC‑A‑AT‑AGGAGAAATCAATATGATCGTTTCTGACATCGAAGCTAACGCCCTCTTAGAGAGCGTCACTAAGTTCCACTGCGGGGTTATCTACGACTACTCCACCGCTGA                                                                                                                                                                                                       >  1:636241/1‑301`  
                                                                                                               `aaggagaagagtataagagacaggaaaAACGTGGAAGCCAAGCGAATGAAAGATGAGGCTCGTAAAGAGGCCACACAGTCACGCGCTCAGGAGATTCGCACCAACGAAATGGCTGACAGTGCATCCACTAAAGTTACCGAGGCTGCCCGTGTGGCAAAACAAGCTCAACAGCTTTACAAATAATTTGAGTAATCAA‑AC‑AGGAGAAACCATTATGATCGTTTCTGACAACGAAGCTAACGCCCTCTTAGAGAGCGTCACTAAGTTCCACTGCGGGGTTATCTACGACTACTCCACCGCTGAG                                                                                                                                                                                                      <  1:261351/274‑1`  
                                                                                                                  `GGCCAACTGCTGGTTCGTATGTACAACGTGGAAGCCAAGCGACTGAACGATGAGGCTCGTAAAGAGGCCACACAGTCACGCGCTCTGGCGATTCGCTCCAACGAACTGGCTGACAGTGCATCCACTAAAGTTACCGAGGCTGCCCGTGTGGCAAACCAAGCTCAACAGCTTTCCAAATTCTTTGAGTAATC‑A‑AT‑AGGAGAAATCAATATGATCGTTTCTGACATCGAAGCTAACGCCCTCTTAGAGAGCGTCACTAAGTTCCACTGCGGGGTTATCTACGACTACTCCACCGCctgtctct                                                                                                                                                                                                  >  1:400281/1‑293`  
                                                                                                                  `GGCCAACTGCTGGTTCGTATGTACAACGTGGAAGCCAAGCGACTGAACGATGAGGCTCGTAAAGAGGCCACACAGTCACGCGCTCTGGCGATTCGCTCCAACGAACTGGCTGACAGTGCATCCACTAAAGTTACCGAGGCTGCCAGTGTGGCAAACCAAGCTCAACAGCTTTCCAAATTCTTTGAGTAATC‑A‑AT‑AGGAGAAATCAATATGATCGTTTATGACATCGAAGCTAACGCCCTCTTAGAGAGCGTCACTAAGTTCCACTGCGGGGTTATCTACGACTACTCCACCGCTGAGTACG                                                                                                                                                                                                  <  1:59634/301‑1`  
                                                                                                                    `gtataagagacagCGTATGTACAACGGGGAAGCCAAGCGACTGAACGATGAGGCTCGTAAAGAGGCCACACAGTCACGCGCTCTGGCGATTCGCTCCAACGAACTGGCTGACAGTGCATCCACTAAAGTTACCGAGGCTGCCCGTGTGGCAAACCAAGCTCAACAGCTTTCCAAATTCTTTGAGTAATC‑A‑AT‑AGGAGAAATCAATATGATCGTTTCTGACATCGAAGCTAACGCCCTCTTAGAGAGCGTCACTAAGTTCCACTGCGGGGTTATCTACGACTACTCCACCGCTGAGTACGTA                                                                                                                                                                                                <  1:483856/288‑1`  
                                                                                                                    `CCAACTGCTGGTTCGTATGTACAACGTGGAAGCCAAGCGACTGAACGATGAGGCTCGTAAAGAGGCCACACAGTCACGCGCTCTGGCGATTCGCTCCAACGAACTGGCTGACAGTGCATCCACTAAAGTTACCGATGCTGCCCGTGTGGCAAACCAAGCTCAACAGCTTTCCAAATTCTTTGAGTAATC‑A‑AT‑AGGAGAAATCAATATGATCGTTTCTGACATCGAAGCTAACGCCCTCTTAGAGAGCGTCACTAAGTTCCACTGCGGGGTTATCTACGACTACTCCACCGCTGAGTACGTA                                                                                                                                                                                                <  1:159904/301‑1`  
                                                                                                                      `AACTGCTGGTTCGTATGTACAACGTGGAAGCCAAGCGACTGAACGATGAGGCTCGTAAAGAGGCCACACAGTCACGAGCTCTGGCGATTCGCTCCAACGAACTGGCTGACAGTGCATCCACTAAAGTTACCGAGGCTGCCCGTGTGGCAAACCAAGCTCAACAGCTTTCCAAATTCTTTGAGTAATC‑A‑AT‑AGGAGAAATCAATATGATCGTTTCTGACATCGAAGCTAACGCCCTCTTAGAGAGCGTCACTAAGTTCCACTGCGGGGTTATCTACGACTACTCCACCGCTGAGTACATAAG                                                                                                                                                                                              <  1:41914/301‑1`  
                                                                                                                        `CTGCTGGTTCGTATGTACAACGTGGAAGCCAAGCGACTGAACGATGAGGCTCGTAAAGAGGCCACACAGTCACGCGCTCTGGCGATTCGCTCCAACGAACTGGCTGACAGTGAATCCACTAAAGTTACCGAGGCTGCCCGTGTGGCAAACCAAGCTCAACAGCTTTCCAAATTCTTTGAGTAATCAA‑AC‑AGGAGAAATCAATATGATCGTTTCTGACATCGAAGCTAACGCCCTCTTAGAGAGCGTCACTAAGTTCCACTGCGGGGTTATCTACGACTACTCCACCGCTGAGctgtctctt                                                                                                                                                                                             >  1:172002/1‑292`  
                                                                                                                          `GCTGGTTCGTATGGACAACGTGGAAGCCAAGCTACTGAACGATGAGGCTCGTAAAGAGGCCACACAGTCACGCGCTCTGGCGATTCGCTCCAACGAACTGGCTGACAGTGCATCCACTAAAGTTACCGAGGCTGCCCGTGTGGCAAACCAAGCTCAACAGCTTTCCAAATTCTTTGAGTAATC‑A‑AT‑AGGAGAAATCAATATGATCGTTTCTGACATCGAAGCTAACGCCCTCTTAGAGAGCGTCACTAAGTTCCACTGCGGGGTTATCTACGACTACTCCACCGCTGAGTACGTAAGCTAC                                                                                                                                                                                          <  1:551584/301‑1`  
                                                                                                                              `GTTCGTATGTACAACGTGGAAGCCAAGCGACTGAACGATGAGGCTCGTAAAGAGGCCACACAGTCACGCGCTCTGGCGATTCGCTCCAACGAACTGGCTGACAGTGCATCCACTAAAGTTACCTAGGCTGCCCGTGTGGCAAACCAAGCTCAACAGCTTTCCAAATTCTTTGAGTAATC‑A‑AT‑ATGAGAAATCAATATGATCGTTTCTGACATCTCAGCTAACGCCCTCTTAGAGAGCGTCACTAAGTTCCACTGCGGGGTTATCTACGACTACTCCACCGCTGAGTACGTAAGCTACCGTC                                                                                                                                                                                      >  1:429331/1‑301`  
                                                                                                                                 `CGTATGTACAACGTGGAAGCCAAGCGACTGAACGATGAGGCTCGTAAAGAGGCCACACAGTCACGCGCTCTGACGATTCGCTCCAACGAACTGGCTGACAGTGCATCCACTAAAGTTACCGAGGCTGCCCGTGTGGCAAACCAAGCTCAACAGCTTTCCAAATTCTTTGAGTAATC‑A‑AT‑AGGAGAAATCAATATGATCGTTTCTGACATCGAAGCTAACGCCCTCTTAGAGAGCGTCACTAAGTTCCACTGCGGGGTTATCTACGACTACTCCACCGCTGAGTACGTAAGCTACCGTCCGA                                                                                                                                                                                   >  1:543152/1‑301`  
                                                                                                                                   `TATGTACAACGTGGAAGCCAAGCGACTGAACGATGAGGCTCGTAAAGAGGCCACACAGTCACGCGCTCTGGCGATTCGCTCCAACGAACTGGCTGACAGTGCATCCACTAAAGTTACCGAGGCTGCCCGTGTGGCAAACCAAGCTCAACAGCTTTCCAAATTCTTTGAGTAATCAA‑AC‑AGGAGAAATCAATATGATCGTTTCTGACATCGAAGCTAACGCCCTCTTAGAGAGCGTCACTAAGTTCCACTGCGGGGTTATCTACGACTACTCCACCGCTGAGTACGTActgtctcttataca                                                                                                                                                                                  >  1:591008/1‑287`  
                                                                                                                                     `TGTACAACGTGGAAGCCAAGCGACTGAACGATGAAGCTCGTAAAGAGGCCACACAGTCACGCCCTCTGGCGATTCGCTCCAACGAACTGGCTGACAGTGCATCCACTAAAGTTACCGAGGCTGCCCGTGTGGCAAACCCAGCTCAACAGCTTTCCAAACTCTTTGAGTAATC‑A‑AT‑AGGAGAAATCAATATGATCGTTTCTGACATCGAAGCTAACGCCCTCTCAGAGAGCGTCACTAAGTTCCACTGCGCGGTTCTCTACGACTACTCTACCGCTGAGTCCGTAAGCTACGGTCCGAGTGA                                                                                                                                                                               >  1:501410/1‑301`  
                                                                                                                                        `ACAAAGTGGAAGCCAAGCGACTGAACGATGAGGCTCGTAAAGAGGCCACACAGTCACGCGCTCTGGCGATTCGCTCCAACGAACTGGCTGACAGTGCATCCACTAAAGTTACCGAGGCTGCCCGTGTGGCAAACCAAGCTCAACAGCTTTCCAAATTCTTTGAGTAATC‑A‑AT‑AGGAGAAATCAATATGATCGTTTCTGACATCGAAGCTAACGCCCTCTTAGAGAGCGTCACTAAGTTCCACTGCGGGGTTATCTACGACTACTCCACCGCTGAGTACGTAAGCTACCGTCCGAGTGACTT                                                                                                                                                                            <  1:67847/301‑1`  
                                                                                                                                           `ACGTGGAAGCCAAGCGACTGAACGATGAGGCTCGTAAAGAGGCCACACAGTCACGCGCTCTGGCGATTCGCTCCAACGAACTGGCTGACAGTGCATCCACTAAAGTTACCGAGGCTGCCCGTGTGGCAAACCAAGCTCAACAGCTTTCCAAATTCTTTGAGTAATC‑A‑AT‑AGGAGAAATCAATATGATCGTTTCTGACATCGAAGCTAACGCCCTCTTAGAGAGCGTCACTAAGTTCCACTGCGGGGTTATCTACGACTACTCCACCGCTGAGTACGTAAGCTACCGTCCGAGTGACTTCct                                                                                                                                                                         >  1:148765/1‑299`  
                                                                                                                                           `ACGTGGAAGCCAAGCGACTGAACGATGAGGCTCGTAAAGAGGCCACACAGTCACGCGCTCTGGCGATTCGCTCCAACGAACTGGCTGACAGTGCATCCACTAAAGTTACCGAGGCTGCCCGTGTGGCAAACCAAGCTCAACAGCTTTCCAAATTCTTTGAGTAATC‑A‑AT‑AGGAGAAATCAATATGATCGTTTCTGACATCGAAGCTAACGCCCTCTTAGAGAGCGTCACTAAGTTCCACTGCGGGGTTATCTACGACTACTCCACCGCTGAGTACGTAAGCTACCGTCCGAGTAAATTCGG                                                                                                                                                                         >  1:220616/1‑301`  
                                                                                                                                                   `GCCAAGCGACTGAACGATGAGGCTCGTAAAGAGGCCACACAGTCACGCGCTCTGGCGATTCGCTCCAACGAACTGGCTGACAGTGCATCCACTAAAGTTACCGAGGCTGCCCGTGTGGCAAACCAAGCTCAACAGCTTTCCAAATTCTTTGAGTAATC‑A‑AT‑AGGAGAAATCAATATGATCGTTTCTGACATCGAAGCTAACGCCCTCTTAGAGAGCGTCACTAAGTTCCACTGCGGGGTTATCTACGACTACTCCACCGCTGAGTACGTAAGCTACCGTCCGAGTGACTTCGGTGCGTATC                                                                                                                                                                 <  1:286481/301‑1`  
                                                                                                                                                       `AGCGACTGAACGATGAGGCTCGTAAAGAGGCCACACAGTCACGCGCTCTGGCGATTCGCTCCAACGAACTGGCTGACAGTGCATCCACTAAAGTTACCGAGGCTGCCCGTGTGGCAAACCAAGCTCAACAGCTTTCCAAATTCTTTGAGTAATC‑A‑AT‑AGGAGAAATCAATATGATCGTTTCTGACATCGAAGCTAACGCCCTCTTAGAGAGCGTCACTAAGTTCCACTGCGGGGTTATCTACGACTACTCCACCGCTGAGTACGTAAGCTACCGTCCGAGTGACTTCGGTGCGTATCTGGA                                                                                                                                                             <  1:249136/301‑1`  
                                                                                                                                                       `AGCGACTGAACGATGAGGCTCGTAAAGAGGCCACACAGTCACGCGCTCTGGCGATTCGCTCCAACGAACTGGCTGACAGTGCATCCACTAAAGTTACCGAGGCTGCCCGTGTGGCAAACCAAGCTCAACAGCTTTCCAAATTCTTTGAGTAATC‑A‑AT‑AGGAGAAATCAATATGATCGTTTCTGACATCGAAGCTAACGCCCTCTTAGAGAGCGTCACTAAGTTCCACTGCGGGGTTATCTACGACTACTCCACCGCTGAGTACGTAAGCTACCGTCCGAGTGACTTCGGTGCGTATCTGGA                                                                                                                                                             <  1:236631/301‑1`  
                                                                                                                                                           `ACTGAACGATGAGGCTCGTAAAGAGGCCACACAGTCACGCGCTCTGGCGATTCGCTCCAACGAACTGGCTGACAGTGCATCCACTAAAGTTACCGAGGCTGCCCGTGTGGCAAACCAAGCTCAACAGCTTTCCAAATTCTTTGAGTAATC‑A‑AT‑AGGAGAAATCAATATGATCGTTTCTGACATCGAAGCTAACGCCCTCTTAGAGAGCGTCACTAAGTTCCACTGCGGGGTTATCTACGACTACTCCACCGCTGAGTACGTAAGCTACCGTCCGAGTGACTTCGGTGCCTGTCTcttatac                                                                                                                                                         >  1:346956/1‑294`  
                                                                                                                                                             `TGAACGATGAGTCTCGGAAAGAGGCCACACAGTCACGCGCTCTGGCGATTCGCTCCAACGAACTGGCTGACAGTGCATCCACTAAAGTTACCGAGGCTGCCCGTGTGGCAAACCAAGCTCAACAGCTTTCCAAATTCTTTGAGTAATCAA‑AC‑AGGAGAAATCAATATGATCGTTTCTGACATCGAAGCTAACGCCCTCTTAGAGAGCGTCACTAAGTTCCACTGCGGGGTTGTCTACGACTACTCCACCGCTGAGTACGTAAGCTACCGTCCGAGTGACTTCGGTGCGTATCTGGATGCGC                                                                                                                                                        <  1:134793/301‑1`  
                                                                                                                                                                                  `AGGCCACACAGTCACGCGCTCTGGCGATTCGCTCCTAAGAACTGGCTGACAGTGCAACCACTAAAGTTACCGAGGCTGCCCGTGTGGCAAACCATGCTCAACAGCTTTCCAAATTCTTTGAGTAATC‑A‑AT‑ATGATAAATCAATATGATCGTTTCTGACATCGAAGCTAACGCCCTCTTAGAGAGCGTCACTAAGTTCCACTGCGGGGATATCTACGACTACTCCACCGCTGTGTACGTAAGCTACCGTCCGAATGACTTAGGTGCGTACCTGGATACGCTGGAAGCCGGAGTTGCACGAGG                                                                                                                                  >  1:105435/1‑301`  
                                                                                                                                                                                   `GGCCACACAGTCACGCGCTCTGGCGATTCGCTCCAACGAACTGGCTGACAGTGCATCCACTAAAGTTACCGAGGCTGCCCGTGTGGCAAACCAAGCTCAACAGCTTTCCAAATTCTTTGAGTAATC‑A‑AT‑AGGAGAAATCAATATGATCGTTTCTGACATCGAAGCTAACGCCCTCTTAGAGAGCGTCACTAAGTTCCACTGCGGGGTTATCTACGACTACTCCACCGCTGAGTACGTAAGCTACCGTCCGAGTGACTTCGGTGCGTATCTGGATGCGCTGGAAGCCGAGGTTGCACGAGGC                                                                                                                                 <  1:51587/301‑1`  
                                                                                                                                                                                       `ACACAGTCACGCGCTCTGGCGATTCGCTCCAACGAACTGGCTGACAGTGCATCCACTAAAGTTACCGAGGCTGCCCGTGTGGCAAACCAAGCTCAACAGCTTTCCAAATTCTTTGAGTAATC‑A‑AT‑AGGAGAAATCAATATGATCGTTTCTGACATCGAAGCTAACGCCCTCTTAGAGAGCGTCACTAAGTTCCACTGCGGGGTTATCTACGACTACTCCACCGCTGAGTACGTAAGCTACCGTCCGAGTGACTTCGGTGCGTATCTGGATGCGCTGGAAGCCGAGGTTGCCCGAGGCGGTC                                                                                                                             >  1:363556/1‑301`  
                                                                                                                                                                                        `aACAGTCACGCGCTCTGGCGATTCGCTCCAACGAACTGGCTGACAGTGCATCCACTAAAGTTACCGAGGCTGCCCGTGTGGCAAACCAAGCTCAACAGCTTTCCAAATTCTTTGAGTAATC‑A‑AT‑AGGAGAAATCAATATGATCGTTTCTGACATCGAAGCTAACGCCCTCTTAGAGAGCGTCACTAAGTTCCACTGCGGGGTTATCTACGACTACTCCACCGCTGAGTACGTAAGCTACCGTCCGAGTGACTTCGGTGCGTATCTGGATGCGCTGGAAGCCGAGGTTGCACGAGGCGGTCT                                                                                                                            <  1:255371/300‑1`  
                                                                                                                                                                                            `GTCACGCGCTCTGGCGATTCGCTCCAACGAACTGGCTGACAGTGCATCCACTAAAGTTACCGAGGCTGCCCGTGTGGCAAACCAAGCTCAACAGCTTTCCAAATTCTTTGAGTAATCAA‑AC‑AGGAGAAATCAATATGATCGTTTCTGACATCGAAGCTAACGCCCTCTTAGAGAGCGTCACTAAGTTCCACTGCGGGGTTATCTACGACTACTCCACCGCTGAGTACGTAAGCTACCGTCCGAGTGACTTCGGTGCGTATCTGGATGCGCTGGAAGCCGAGGTTGCACGAGGCGGTCTTAT                                                                                                                         <  1:36900/301‑1`  
                                                                                                                                                                                             `tccCGCGCTCTGCGTATTCGCTCCACGGAACTTGCTGCCAGTGCATCCGCTAAAGTTACCGAGGCTGCCCGTGTGGCAAACCAAGCTCAACAGCTTTCCAAATTCATTGAGTAATC‑A‑AT‑AGGCGAAAACAATATGATCGTTTCTGACATCGAAGCTAACGCCCTCTTAGAGGGTGTCGCTAAGTTCCACTGCGGGGTTATCTACGACTACTCCACCGCTGAGTACGTAAGCTACCGTCCGAGTGACTTCGGTGCGTATCTGGATGCGCTGGAAGCCGAGGTTGCACGAGGCAGTCTTATTG                                                                                                                       <  1:435106/298‑1`  
                                                                                                                                                                                             `TCACGCGCTCTGGCGATTCGCTCCAACGAACTGGCTGACAGTGCATCCCCTAAAGTTACCGAGGCTGCCCGTGTGGCAAACCAAGCTCTACAGCTTTCCAAATTCTTTGAGTAATC‑A‑AT‑AGGAGAAATCAATATGATCGTTTCTGACATCGAAGCTAACGCCCTCTTAGAGAGCGTCACTAAGTTCCACTGCGGGGTTATCTACGACTACTCCACCGCTGAGTACGTAAGCTACCGTCCGAGTGACTTCGGTGCGTATCTGGATGCGCTGGAAGCCGAGGTTGCACGAGGCGGTCTTATTG                                                                                                                       <  1:509031/301‑1`  
                                                                                                                                                                                             `TCACGCGCTCTGGCGATTCGCTCCAACGAACTGGCTGACAGTGCATCCACTAAAGTTACCGAGGCTGCCCGTGTGGCAAACCAAGCTCAACAGCTTTCCAAATTCTTTGAGTAATC‑A‑AT‑AGGAGAAATCAATATGATCGTTTCTGACATCGAAGCTAACGCCCTCTTAGAGAGCGTCACTAAGTTCCACTGCGGGGTTATCTACGACTACTCCACCGCTGAGTACGTAAGCTACCGTCCGAGTGACTTCGGTGCGTATCTGGATGCGCTGGAAGCCGAGGTTGCACGAGGCGGTCTTATTG                                                                                                                       <  1:557304/301‑1`  
                                                                                                                                                                                              `aacacaCTCTGGCGATTCGCTCCAAGGAACTGGCTGACAGTGCATCCACTAAAGGTACCGAGGCTGCCCGTGTGGCAAACCAAGCTCAACAGCTTTCCAAATTCTTTGAGTAATCAA‑AC‑AGGAGAAATCAATATGATCGTTTCTGACATCGAAGCTAACGCCCTCTTAGAGAGCGTCACTAAGTTCCACTGCGGGGTTATCTACGACTACTCCACCGCTGAGTACGTAAGCTACCGTCCGAGTGACTTCGGTGCGTATCTGGATGCGCTGGAAGCCGAGGTTGCACGAGGCGGTCTTATTG                                                                                                                       <  1:642391/295‑1`  
                                                                                                                                                                                              `CACGCGCTCTGGCGATTCGCTCCAACGAACTGGCTGACAGTGCATCCACTAAAGTTACCGAGGCTGCCCGTGTGGCAAACCAAGCTCAACAGCTTTCCAAATTCTTTGAGTAATC‑AGAC‑AGGAGAAATCAATATGATCGTTTCTGACATCGAAGCTAACGCCCTCTTAGAGAGCGTCACTAAGTTCCACTGCGGGGTTATCTACGACTACTCCACCGCTGAGTACGTAAGCTACCGTCCGAGTGACTTCGGTTCGTATCTGGCTTCGCTGGAAGCCGAGGTTGCACGAGGCGGTCTTATTG                                                                                                                       <  1:615077/301‑1`  
                                                                                                                                                                                                                 `CTCCAACGAACTGGCTGACAGTGCATCCACTAAAGTTACCGAGGCTGCCCGTGTGGCAAACCAAGCTCAACAGCTTTCCAAATTCTTTGAGTAATC‑A‑AT‑AGGAGAAATCAATATGATCGTTTCTGAAATCGAAGCTAACGACCTCTTAGAGAGCGTCACTAAGTTCCACTGCGGGGTTATCTACGACTACTCCACCGCTGAGTACGTAAGCTAACGTCCGAGTGACTTCGGTGCGTATCTGGATGCGCTGGAAGCCGAGGTTGCACGAGGCGGTCTTATTGTGTTCCACAACGGTCACAAG                                                                                                   <  1:184563/301‑1`  
                                                                                                                                                                                                                        `aAACTGGCTGACAGTGCATCCACTAAAGTTACCGAGGCTGCCCGTGTGGCAAACCAAGCTCAACAGCTTTCCAAATTCTTTGAGTAATC‑A‑AT‑AGTAGAAATCAATATGATCGTTTCTGACATCGAAGCTAACGCCCTCTTAGAGAGCGTCACTAAGTTCCACTGCGGGGTTATCTACGACTACTCCACCGCTGAGTACGTAAGCTACCGTCCGAGTGACTTCGGTGCGTATCTGGATGCGCTGGAAGCCGAGGTTGCACGAGGCGGTCTTATTGTGTTCCACAACGGTCACAAGTATGACG                                                                                            <  1:457944/300‑1`  
                                                                                                                                                                                                                         `AACTTGCTGACAGTGCATCCACTAAAGTTACCGAGGCTGCCCGTGTGGCAAACCAAGCTCAACAGCTTTCCAAATTCTTTGAGTAATC‑A‑AT‑AGGAGAAATCAATATGATCGTTTCTGACATCGATGCTAACGCCCTCTTAGAGTGCGTCACTAAGTTCCACTGCGGGGTTATCTACGACTACTCCACCGCTGAGTACGTAAGCTACCGTCCGAGTGACTTCGGTGCGTATCTGGATGCGCTGGAAGCCGAGGTTGCACGAGGCGGTCTTATTGTGTTCCACAACGGTCACAAGTATGAAGT                                                                                           <  1:520626/301‑1`  
                                                                                                                                                                                                                         `AACTGGCTGACAGTGCATCCACCAAAGTTACCGAGGCTGCCCGTGTGGCAAACCAAGCTCAACAGCTTTCCAAATTCTTTGAGTAATCAA‑AG‑AGGAGAAATCAATATGTTCGTTTCTGACATCGAAGCTAACGCCCTCTTAGAGAGCGTCACTAAGTTCCACTGCGGGGTTATCTACGACTACTCCACCGCTGAGTACGTAAGCTACCGTCCGAGTGACTTCGGTGCGTATCTGGATGCGCTGGAAGCCGAGGTTGCACGAGGCGGTCTTATTGTGTTCCACAACGGTCACAAGTATGACG                                                                                            <  1:190481/301‑1`  
                                                                                                                                                                                                                          `ACTGGCTGACAGGGCATCCACTAAAGTTACCGAGGCTGCCCGTGTGGCAAACCAAGCTCAACAGCTTTCCAAATTCTTTGAGTAATC‑A‑AT‑AGGAGAAATCAATAAGATCGTTTCTGACATCGAAGCTAACGCCCTCTTAGAGAGCGTCACTAAGTTCCACTGCGGGGTTATCTACGACTACTCCACCGCTGAGTACGTAAGCTACCGTCCGAGTGACTTCGGTGCGTATCTGGATGCGCTGGAAGCCGAGGTTGCACGAGGCGGTATTATTGTGTTCCACAACGGTCACAAGTATGACGTT                                                                                          <  1:620211/301‑1`  
                                                                                                                                                                                                                                 `aaagagacagCCACTAAAGTTACCGAGGCTGCCCGTGTGGCAAACCAAGCTCAACAGCTTTACAAATTCGTTGAGTAATC‑A‑AT‑AGGAGAAATCAATATGATCGTTTCTGACATCGAAGCTAACGCCCTCTTAGAGAGCGTCACTAAGTTCCACTGCGGGGTTATCTACGACTACTCCACCGCTGAGTACGTAAGCTACCGTCCGAGTGACTTCGGTGCGTATCTGGATGCGCTGGAAGCCGAGGTTGCACGAGGCGGTCTTATTGTGTTCCACAACGGTCACAAGTATGACGTTCCTGCAT                                                                                   <  1:490439/291‑1`  
                                                                                                                                                                                                                                 `GACAGTGCATCCACTAAAGTTACCGAGGCTGCCCGTGTGGCAAACCAAGCTCAACAGCTTTCCAAATTCTTTGAGTAATC‑A‑AT‑AGGAGAAATCAATATGATCGTTTCTGACATCGAAGCTAACGCCCTCTTAGAGAGCGTCACTAAGTTCCACTGCGGGGTTATCTACGACTACTCCACCGCTGAGTACGTAAGCTACCGTCCGAGTGACTTCGGTGCGTATCTGGATGCGATGGAAGCCGAGGTTGCACGAGGCGGTCTTATTGTGTTCCACAACGGTCACAAGTATGACGTTCCTGCAT                                                                                   <  1:477013/301‑1`  
                                                                                                                                                                                                                                 `GACAGTGCATCCACTAAAGTTACCGAGGCTGCCCGTGTGGCAAACCAAGCTCAACAGCTTTCCAAATTCTTTGAGTAATC‑A‑AT‑AGGAGAAATCAATATGATCGTTTCTGACATCGAAGCTAACACCCTCTTAGAGAGCGTCACTAAGTTCCACTGCGGGGTTATCTACGACTACTCCACCGCTGAGTACGTAAGCTACCGTCCGAGTGACTTCGGTGCGTATCTGGATGCGCTGGAAGCCGAGGTTGCACGAGGCGGTCTTATTGTGTTCCACAACGGTCACAAGTATGACGTTCCTGCAT                                                                                   <  1:524732/301‑1`  
                                                                                                                                                                                                                                 `GACAGTGCATCCACTAAAGTTACCGAGGCTGCCCGGGTGGCAAACCAAGCTCAACAGCTTTCCAAATTCTTTGAGTAATC‑A‑AT‑AGGAGAAATCAATATGATCGTTTCTGACATCGAAGCTAACGCCCTCTTAGAGAGCGTCACTAAGTTCCACTGCGGGGTTATCTACGACTACTCCACCGCTGAGTACGTAAGCTACCGTCCGAGTGACTTCGGTGCGTATCTGGATGCGCTGGAAGCCGAGGTTGCACGAGGCGGTCTTATTGTGTTCCACAACGGTCACAAGTATGACGTTCCTGCAT                                                                                   <  1:796/301‑1`  
                                                                                                                                                                                                                                 `GACAGGTCATCCACTAAAGTTACCGAGGCTGCCCGTGTGGAAAACCAAGCTCAACAGCTTTCCAAATTCTTTGAGTAATC‑A‑AT‑AGGAGAAATCAATATGATCGTTTCTGACATCGAAGCTAACGCCCTCTTAGAGGGCGTCACTAAGTTCCACTGCGGGGTTATCTACGACTACTCCACCGCTGAGTACGTAAGCTACCGTCCGAGTGACTTCGGTGCGTATCTGGATGCGCTGGAAGCCGAGGTTGCACGAGGCGGTCTTATTGTGTTCCACAACGGTCACAAGTATGACGTTACTGCAT                                                                                   <  1:423147/301‑1`  
                                                                                                                                                                                                                                                     `TACCGAGGCTGCCCGTGTGGCAAACCAAGCTCAACAGCTTTCCAAATTCTTTGAGTAATC‑A‑AT‑AGGAGAAATCAATATGATCGTTTCTGACATCGAAGCTAACGCCCTCTTAGAGAGCGTCACTAAGTTCCACTGCGGGGTTATCTACGACTACTCCACCGCTGAGTACGTAAGCTACCGTCCGAGTGACTTCGGTGCGTATCTGGATGCGCTGGAAGCCGAGGTTGCACGCGGCGGTCTTATTGTGTTCCACAACGGTCACAAGTATGACGTTCCTGCATTGACCAAACTGGCAAAGTTG                                                               >  1:154596/1‑301`  
                                                                                                                                                                                                                                                          `gatgtgtataagagacagACCAAGCTCAACAGCTTTCCAAATTCTTTGAGTAATC‑A‑AT‑AGGAGAAATCAATATGATCGTTTCTGACATCGAAGCTAACGCCCTCTTAGAGAGCGTCACTAAGTTCCACTGCGGGGTTATCTACGACTACTCCACCGCTGAGTACGTAAGATACCGTCCGAGTGACTTCGGTGCGTATCTGGATGCGCTGGAAGCCGAGGTTGCACGAGGCGGTCTTATTGTGTTCCACAACGGTCACAAGTATGACGTTCCTGCATTGACCAAACTGGCAAAGTTGCAATT                                                          <  1:446977/283‑1`  
                                                                                                                                                                                                                                                                   `GTGTGGCAAACCAAGCTCAACAGCTTTCCAAATTCTTTGAGTAATC‑A‑AT‑AGGAGAAATCAATATGATCGTTTCTGACATCGAAGCTAACGCCCTCTTAGAGAGCGTCACTAAGTTCCACTGCGGGGTTATCTACGACTACTCCACCGCTGAGTACGTAAGCTACCGTCCGAGTGACTTCGGTGCGTATCTGGATGCGCTGGAAGCCGAGGTTGCACGAGGCGGTCTTATTGTGTTCCACAACGGTCACAAGTATGACGTTCCTGCATTGACCAAACTGGCAAAGTTGCAATTGAACCGAGA                                                 <  1:225562/301‑1`  
                                                                                                                                                                                                                                                                               `AAGCTCAACAGCTTTCCAAATTCTTTTAGTAATCAA‑AC‑AGGAGAAACCATTATGATCGTTTCTGACATCGAATCTAACGCCCTCTTAGAGAGCGTCACTAAGTTCCACTTCGGGGTTATCTACGACTACTCCACCGCTGAGTACGTAAGCTACCGTCCGAGTGACTTCGGTGCGTATCTGGATGCGCTGGAAGCCGAGGTTGCACGAGGCGGTCTTATTGTGTTCCACAACGGTCACAAGTATGACGTTCCTGCATTGACCAAACTGGCAAAGTTGCAATTGAACCGAGAGTTCCACCTTC                                      <  1:279755/301‑1`  
                                                                                                                                                                                                                                                                                 `GCTCAACAGCTTTCCAAATTCTTTGAGTAATC‑A‑AT‑AGGAGAAATCAATATGATCGTTTCTGACATCGAAGCTAACGCCCTCTTAGAGAGCGTCACTAAGTTCCACTGCGGGGTTATCTACGACTACTCCACCGCTGAGTACGTAAGCTACCGTCCGAGTGACTTCGGTGCGTATCTGGATGCGCTGGAAGCCGAGGTTGCACGAGGCGGTCTTATTGTGTTCCACAACGGTCACAAGTATGACGTTCCTGCATTGACCAAACTGGCAAAGTTGCAATTGAACCGAGAGTTCCACCTTCCTC                                   >  1:178620/1‑301`  
                                                                                                                                                                                                                                                                                             `TCCAAATTCTCTGAGTAATCAA‑AC‑AGGAGAAATCAATATGATCGTTTCTGACATCGAAGCTAACGCCCTCTTAGAGAGCGTCACTAAGTTCCACTGCGGGGTTATCTACGACTACTCCACCGCTGAGTACGTAAGCTACCGTCCGAGTGACTTCGGTGCGTATCTGGATGCGCTTGAAGCCGAGGTTGCACGAGGCGGTCTTATTGTGTTCCACAACGGTCACAAGTATGACGTTACTGCATTGACCAAACTGGCCAAGTTGCAATTGAACCGAGCGTTCCACCTTCCTCGTGAGAACTGT                        >  1:327400/1‑301`  
                                                                                                                                                                                                                                                                                                   `TTCTTTGAGTAATC‑A‑AT‑AGGAGAAATCAATATGATCGTTTCTGACATCGAAGCTAACGCCCTCTTAGAGAGCGTCACTAAGTTCCACTGCGGGGTTATCTACGACTACTCCACCGCTGAGTACGTAAGCTACCGTCCGAGTGACTTCGGTGCGTATCTGGATGCGCTGGAAGCCGAGGTTGCACGAGGCGGTCTTATTGTGTTCCACAACGGTCACAAGTATGACGTTCCTGCATTGACCAAACTGGCAAAGTTGCAATTGAACCGAGAGTTCCACCTTCCTCGTTAGAACTGTATTctgt                 >  1:648637/1‑297`  
                                                                                                                                                                                                                                                                                                    `TCTATGAGTAATC‑A‑AT‑AGGAGTAATCAAGAGGATCGTTTCTGACAGCGAAGCTAACGCCCTCTTAGAGAGCGTCACTAAGTTCCACTGCGGGGTTATCTACGACTACTCCACCGCTGAGTACGTAAGCTACCGTCCGAGTGACTTCGGTGCGTATCTGGATGCGCTGGAAGCCGATGTTGCACGAGGCGGTCTTATTGTGTTCCACAACGGTCACAAGTATGACGTTCCTGCATTGACCAAACTGGCAAAGTTGCAATTGAACCGAGAGTTCCACCTTCCTCGTGAGAACTGTATTGACAC                <  1:666847/301‑1`  
                                                                                                                                                                                                                                                                                                     `agacagGTAATC‑A‑AT‑AGGAGAAATCAATATGATCTGTTCTGACATCGAAGCTAACGCCCTCGTAGAGAGCGTCACTAAGTTCCACTGCGGGGTTATCTACGACTACTCCACCGCTGAGTACGTAAGCTACCGTCCGAGTGACTTCGGTGCGTATCTGGATGCGCTGGAAGCCGAGGTTGCACGAGGCGGTCTTATTGTGTTCCACAACGGTCACAAGTATGACGTTCCTGCATTGACCAAACTGGCAAAGTTGCAATTGAACCGAGAGTTCCACCTTCCTCGTGAGAACTGTATTGACACC               <  1:609475/295‑1`  
                                                                                                                                                                                                                                                                                                         `GAGTAATC‑A‑AT‑AGGAGAAATCAATATGATCGTTTCTGACATCGAAGCTAACGCCCTCTTAGAGAGCGTCACTAAGTTCCACTGCGGGGTTATCTACGACTACTCCACCGCTGAGTACGTAAGCTACCGTCCGAGTGACTTCGGTGCGTATCTGGATGCGCTGGAAGCCGAGGTTGCACGAGGCGGTCTTATTGTGTTCCACAACGGTCACACGTATGACGTTCCTGCATTGACCAAACTGGCAAAGTTGCAATTGAACCGAGAGTTCCACCTTCCTCGTGAGAAATGTATTGACACCtgtc           >  1:142292/1‑297`  
                                                                                                                                                                                                                                                                                                         `GAGTAATC‑A‑AT‑AGGAGAAATCAATATGATCGTTTCTGACATCGAAGCTAACGCCCTCTTAGAGAGCGTCACTAAGTTCCACTGCGGGGTTATCTACGACTACTCCACCGCTGAGTACGTAAGCTACCGTCCGAGTGACTTCGGTGCGTATCTGGATGCGCTGGAAGCCGAGGTTGCACGAGGCGGTCTTATTGTGTTCCACAACGGTCACAAGTATGACGTTCCTGCATTGACCAAACTGGCAAAGTTGCAATTGAACCGAGAGTTCCACCTTCCTCGTGAGAACTGTATTTACACtgtcc           >  1:359440/1‑296`  
                                                                                                                                                                                                                                                                                                            `cagataA‑AT‑AGGAGAAATCAATATGATCGTTTCTGACATCGAAGCTAACGCCCTCTGAGAGAGCGTCACTAAGTTCCACTGCGGGGTTATCTACGACTACTCCACCGCTGAGTACGTAAGCTACCGTCCTAGTGACTTCGGTGCGTATCTGGATGCGCTGGAAGCCGAGGTTGCACGAGGCGGTCTTATTGTGTTCCACAACGGTCACAAGTATGACGTTCCTGCATTGACCAAACTGGCAAAGTTGCAATTGAACCGAGAGTTCCACCTTCCTCGTGAGAACTGTATTGACACCCTTGTG         <  1:62525/295‑1`  
                                                                                                                                                                                                                                                                                                            `acagC‑A‑AT‑AGGAGAAATCAATATGATCGTTTCTGACATCGAAGCTAACGCCCTCTTAGAGAGCGTCACTAAGTTCCACTGCGGGGTTATCTACGACTACTCCACCGCTGAGTACGTAAGCTACCGTCCGAGTGACTTCGGTGCGTATCTGGATGCGCTGGAAGCCGAGGTTGCACGAGGCGGTCTTATTGTGTTCCACAACGGTCACAAGTATGACGTTCCTGCATTGACCAAACTGGCAAAGTTGCAATTGAACCGAGAGTTCCACCTTCCTCGTGAGAACTGTATTGACACCCTTGTGT        <  1:529968/297‑1`  
                                                                                                                                                                                                                                                                                                             `AATC‑A‑AT‑AGGAGAAATCAATATGATCGTTTCTGACATCGAAGCTAACGCCCTCTTAGAGAGCGTCCCTAAGTTCCACTGCGGGGTTATCTACGACTACTCCACCGCTGAGTACGTAAGCTACCGTCCGAGTGACTTCGGTGCGTATCTGGATGCGCTGGAAGCCGAGGTTGCACGAGGCGGTCTTATTGTGTTCCACAACGGTCACAAGTATGACGTTCCTGCATTGACCAAACTGGCAAAGTTGCAATTGAACCGAGAGTTCCACCTTCCTCGTGAGAACTGTATTGACACACTTGTGTT       >  1:215189/1‑301`  
                                                                                                                                                                                                                                                                                                              `ATC‑A‑AT‑AGGAGAAATCAATATGATCGTTTCTGACATCGAAGCTAACGCCCTCTTAGAGAGCGTCACTAAGTTCCACTGCGGGGTGATCTACGACTACTCCACCGCTGAGTACGTAAGCTACCGTCCGAGTGACTGCGGTGCGTATCTGGATGCGCTGGAAGCCGAGGGTGCACGAGGCGGTCGTATTGTGGGCAACAACGGGCACAAGTATGACGATCGGGAATAGACCACACAGGCAAAGTGGCAATGGAACCGAGAGTTCCACCTTCCTCGTGAGGACTGTATTGACACACTgtctctt      >  1:29230/1‑294`  
                                                                                                                                                                                                                                                                                                               `TC‑A‑AT‑AGGAGAAATCAATATGATCGTTTCTGACATCGAAGCTAACGCCCTCTTAGAGAGCGTCACTAAGTTCCACTTCGGGGTTATCTACGACTACTCCACCGCTGAGTACGTAAGCTACCGTCCGAGTGACTTCGGTGCGTATCTGGATGCGCTGGAAGCCGAGGTTGCACGAGGCGGTCTTATTGTGTTCCACAACGGTTACAAGTATGACGTTCCTGCATTGACCAAACTGGCAAAGTTGCAATTTAACCGAGAGTTCCACCTTCCTCGTGAGAACctgtctcttatacacatctccc     >  1:74492/1‑279`  
                                                                                                                                                                                                                                                                                                               `TC‑A‑AT‑AGGAGAAATCAATATGATCGTTTCTGACATCGAAGCTAACGCCCTCTTAGAGAGCGTCACTAAGTTCCACTGCGGGGTTATCTACGACTACTCCACCGCTGAGTACGTAAGCTACCGTCCGAGTGACTTCGGTGCGTATCTGGATGCGCTGGAAGCCGAGGTTGCACGAGGCGGTCTTATTGTGTTCCACAACGGTCACAAGTATGACGTTCCTGCATTGACCAAACTGGCAAAGTTGCAATTGAACCGAGAGTTCCACCTTCCTCGTGAGAACTGTATTGACACCCTTGTGTTGT     >  1:411227/1‑301`  
                                                                                                                                                                                                                                                                                                                `C‑A‑AT‑AGGAGAAATCAATATGATCGTTTCTGACATCGAAGCTAACGCCCTCTTAGAGAGCGTCACTAAGTTCCACTGCGGGGTTATCTACGACTACTCCACCGCTGAGTACGTAAGCTACCGTCCGAGTGACTTCGGTGCGTATCTGGATGCGCTGGAAGCCGAGGTTGCACGAGGCGGTCTTATTGTGTTCCACAACGGTCACAAGTATGACGTTCCTGCATTGACCAAACTGGCAAAGTTGCAATTGAACCGAGAGTTCCACCTTCCTCGTGAGAACTGTATTTACACCCTTGTGTctgt    >  1:388738/1‑297`  

                                                                                                                                                                                                                                                                                                                                                                                                                                                                                                                                                                                                                                    
`CTGACAGGATTCTTGATGACTTTCCAGACGACTACGAGAAGTTTCGCTGGAGAGTCCCATTCTAATACGACTCACTAAAGGAGACACACCATGTTCAAACTGATTAAGAAGTTAGGCCAACTGCTGGTTCGTATGTACAACGTGGAAGCCAAGCGACTGAACGATGAGGCTCGTAAAGAGGCCACACAGTCACGCGCTCTGGCGATTCGCTCCAACGAACTGGCTGACAGTGCATCCACTAAAGTTACCGAGGCTGCCCGTGTGGCAAACCAAGCTCAACAGCTTTCCAAATTCTTTGAGTAATC‑A‑A‑‑‑‑‑‑‑‑‑‑‑‑‑‑‑‑‑‑‑‑‑‑‑‑‑‑‑‑‑‑‑‑‑‑‑‑‑‑‑‑‑‑‑‑‑‑‑‑‑‑‑‑‑‑‑‑‑‑‑‑‑‑‑‑‑‑‑‑‑‑‑‑‑‑‑‑‑‑‑‑‑‑‑‑‑‑‑‑‑‑‑‑‑‑‑‑‑‑‑‑‑‑‑‑‑‑‑‑‑‑‑‑‑‑‑‑‑‑‑‑‑‑‑‑‑‑‑‑‑‑‑‑‑‑‑‑‑‑‑‑‑‑‑‑‑‑‑‑‑‑‑‑‑‑‑‑‑‑‑‑‑‑‑‑‑‑‑‑‑‑‑‑‑‑‑‑‑‑‑‑‑‑‑‑‑‑‑‑‑‑‑‑‑‑‑‑‑‑‑‑‑‑‑‑‑‑‑‑‑‑‑‑‑‑‑‑‑‑‑‑‑‑‑‑‑‑‑‑‑‑‑‑‑‑‑‑‑‑‑‑‑‑‑‑‑‑‑‑‑‑‑‑‑‑‑‑‑‑‑‑‑‑‑‑‑‑‑‑‑‑‑‑‑‑‑‑‑‑‑‑‑‑‑‑‑‑‑‑‑‑‑‑‑‑‑‑‑‑‑‑‑  >  NC_001604.1/13262‑13568`  
`‑‑‑‑‑‑‑‑‑‑‑‑‑‑‑‑‑‑‑‑‑‑‑‑‑‑‑‑‑‑‑‑‑‑‑‑‑‑‑‑‑‑‑‑‑‑‑‑‑‑‑‑‑‑‑‑‑‑‑‑‑‑‑‑‑‑‑‑‑‑‑‑‑‑‑‑‑‑‑‑‑‑‑‑‑‑‑‑‑‑‑‑‑‑‑‑‑‑‑‑‑‑‑‑‑‑‑‑‑‑‑‑‑‑‑‑‑‑‑‑‑‑‑‑‑‑‑‑‑‑‑‑‑‑‑‑‑‑‑‑‑‑‑‑‑‑‑‑‑‑‑‑‑‑‑‑‑‑‑‑‑‑‑‑‑‑‑‑‑‑‑‑‑‑‑‑‑‑‑‑‑‑‑‑‑‑‑‑‑‑‑‑‑‑‑‑‑‑‑‑‑‑‑‑‑‑‑‑‑‑‑‑‑‑‑‑‑‑‑‑‑‑‑‑‑‑‑‑‑‑‑‑‑‑‑‑‑‑‑‑‑‑‑‑‑‑‑‑‑‑‑‑‑‑‑‑‑‑‑‑‑‑‑‑‑‑‑‑‑‑‑‑‑‑‑‑‑‑‑‑‑‑‑‑‑‑‑‑‑‑‑‑‑‑‑‑‑‑‑‑taatc‑a‑aT‑AGGAGAAATCAATATGATCGTTTCTGACATCGAAGCTAACGCCCTCTTAGAGAGCGTCACTAAGTTCCACTGCGGGGTTATCTACGACTACTCCACCGCTGAGTACGTAAGCTACCGTCCGAGTGACTTCGGTGCGTATCTGGATGCGCTGGAAGCCGAGGTTGCACGAGGCGGTCTTATTGTGTTCCACAACGGTCACAAGTATGACGTTCCTGCATTGACCAAACTGGCAAAGTTGCAATTGAACCGAGAGTTCCACCTTCCTCGTGAGAACTGTATTGACACCCTTGTGTTGTCAC  >  NC_001604.1/14339‑14638`

  

Alignment Legend

Aligned base mismatch/match (shaded by quality score): `ATCG/ATCG` `< 3 ≤` `ATCG/ATCG` `< 10 ≤` `ATCG/ATCG` `< 22 ≤` `ATCG/ATCG` `< 34 ≤` `ATCG/ATCG` `< 38 ≤` `ATCG/ATCG`

Unaligned base: `atcg`    Masked matching base: `atcg`    Alignment gap: `‑`    Deleted base: `‑`

Reads not counted as support for junction

read\_name Not counted due to insufficient overlap past the breakpoint.

read\_name Not counted due to not crossing MOB target site duplication.
